# 

**Published:** 1900-01

**Authors:** 


### BOOKS RECEIVED.

Musser's Medical Diagnosis. A Practical Treatise on Medical Diagnosis. For the use of students and practitioners. By John H. Musser, M. D., Professor of Clinical Medicine, University of Pennsylvania, Philadelphia. New (third) edition, thoroughly revised. Octavo, 1082 pages, with 253 engravings and 48 full-page colored plates. Just ready. Cloth, $6.00 net; leather, $7.00 net.

Progressive Medicine. Volume IV. A Quarterly Digest of Advances, Discoveries and Improvements in the Medical and Surgical Sciences. Edited by Hobart Amory Hare, M. D., Professor of Therapeutics and Materia Medica in the Jefferson Medical College of Philadelphia. Octavo, handsomely bound in cloth, 398 pages, fifty-one engravings and five plates. Philadelphia and New York : Lea Brothers & Co.

Refraction and How to Refract. Including Sections on Optics, Retinoscopy, the Fitting of Spectacles and Eye glasses, etc, By James Thorington, A. M., M. D., Adjunct Professor of Ophthalmology in the Philadelphia Polyclinic and College for Graduates in Medicine ; Assistant Surgeon at Wills' Eye Hospital ; Associate Member of the American Ophthalmological Society ; Fellow of the College of Physicians of Philadelphia ; Member of the American Medical Association ; Ophthalmologist to the Elwyn and the Vineland Training Schools for Feeble-minded Children ; Resident Physician and Surgeon, Panama Railroad Co., at Colin (Aspinwall), Isthmus of Panama, 1882–1889, etc. Two hundred illustrations, thirteen of which are colored. Octavo, 301 pages. Cloth, $1.50 net. Philadelphia : P. Blakiston's Son & Co., 1012 Walnut Street.

A Manual of Modern Gastric Methods, Chemical, Physical and Therapeutical. By A. Lockhart Gillespie, M. D., F. R. C. P. E., F. R. S., E., Lecturer on Materia Medica and Therapeutics in the School of Medicine of the Royal College, Edinburgh ; Post-Graduate Lecturer on Modern Gastric Methods, Edinburgh, etc., etc. With a chapter upon the mechanical methods used in young children, by John Thomson, M. D., F. R. C. P Ed., Assistant Physician, Royal Hospital for Sick Children, Edinburgh. New York : William Wood & Co. 1899.

Transactions of the American Ophthalmological Society. Thirty-second annual meeting, Pequot House, New London, Conn., July 18, 1899. Vol. VII., Part II. Published by the Society. New Bedford, Mass.: Mercury Publishing Co., Printers. 1899.

Transactions of the American Gynecological Society. Volume XXIV. For the year 1899. Edited by J. Riddle Goffe, M. D., Secretary. Philadelphia : William J. Dornan, Printer. 1899.

A Manual of Diseases of the Eye. By Edward Jackson, A. M., M. D., Emeritus Professor of Diseases of the Eye in the Philadelphia Polyclinic ; Member of the American Ophthalmological Society, etc., etc. With 178 illustrations and two colored plates. Philadelphia : W. B. Saunders. 1900.

Report of the Commissioner of Education for the Year 1897-98. Volume I. Containing Part I. Washington : Government Printing Office. 1899.

